# Pediatric and Youth Traffic-Collision Injuries in Al Ain, United Arab Emirates: A Prospective Study

**DOI:** 10.1371/journal.pone.0068636

**Published:** 2013-07-04

**Authors:** Michal Grivna, Hani O. Eid, Fikri M. Abu-Zidan

**Affiliations:** 1 Institute of Public Health, College of Medicine and Health Sciences, UAE University, Al Ain, United Arab Emirates; 2 Trauma Group, Department of Surgery, College of Medicine and Health Sciences, UAE University, Al Ain, United Arab Emirates; Iran University of Medical Sciences, Islamic Republic of Iran

## Abstract

**Aim:**

To study the mechanism of road traffic collisions (RTC), use of safety devices, and outcome of hospitalized pediatric and youth RTC injured patients so as to give recommendations regarding prevention of pediatric RTC injuries.

**Methods:**

All RTC injured children and youth (0–19-year-olds) who were admitted to Al Ain City’s two major trauma centers or who died after arrival to these centers were prospectively studied from April 2006 to October 2007. Demography of patients, road-user and vehicle types, crash mechanism, usage of safety devices, injured body regions, injury severity, Revised Trauma Score, Glasgow Coma Scale, intensive care unit admissions, hospital stay and mortality were analyzed.

**Results:**

245 patients were studied, 69% were vehicle occupants, 15% pedestrians, 9% motorcyclists and 5% bicyclists. 79% were males and 67% UAE citizens. The most common mechanism of RTC was rollover of vehicle (37%) followed by front impact collision (32%). 32 (13%) of vehicle occupants were ejected from car. 63% of ejected occupants and 70% of motorcyclists sustained head injuries. Only 2% (3/170) vehicle passengers used seatbelts and 13% (3/23) motorcyclists a helmet.

**Conclusions:**

Male drivers and UAE nationals were at high risk of RTC as drivers and as motorcyclists. Ejection rate was high because safety restraint use was extremely low in our community. More education and law enforcement focusing especially on car/booster seat use is needed.

## Introduction

United Arab Emirates (UAE) is a rapidly developing country in economic transition with an increasing population and a growing number of cars and vulnerable road users. In the time of the study, UAE population was approximately 5 million [1]. Traffic-related collision is a major cause of death among children and youth in the UAE, causing on average 103.7 deaths per year (2000–2008) with incidence mortality 13.6 per 100.000 population [2]. These injuries impose a great burden on the affected persons, families and health care facilities. Although risk factors of road traffic collisions are well-described, they can vary in different settings. During the cognitive development process of children their ability to make safe decisions on the roads is not mature [3]. Young children may unintentionally take risks because of lacking of appropriate skills. On the other hand, older children and adolescents may be actively seeking out for risk [4]. Incidence and prevention of road traffic injuries, including creating safe road environments and using safety restraints, have been extensively studied [5]. Despite legislation and increasing law enforcement, there is still low use of safety restraints in our community [2], [6], [7].

There has been recently an attempt by Health Authority Abu Dhabi to establish an injury surveillance system based on Emergency Department visits [2]. Nevertheless, it is still in the implementation stage and data were not yet published. A prospective hospital based study can assess more severe pediatric injuries. We have previously reported the high frequency of traffic-related head injury among UAE children and youth and suggested that the use of safety restraints was low although these specific data were not available in that study [8]. Hospitalized trauma patients in Al Ain are exclusively admitted to two hospitals. Al Ain Hospital has 412 beds and provides a wide range of general and specialist clinical services [9], whereas Tawam Hospital is a highly specialized tertiary care center with 468 beds [10]. They are serving Al Ain City with a population of about 460,000 inhabitants [11]. Al Ain is the largest desert city in the eastern district of Abu Dhabi and one of the four largest in in the UAE. It is situated in the Abu Dhabi Emirate, which includes over 85% of the country’s surface area and nearly 40% of the population [12].

There is lack of data on traffic-related injuries among children and youth in the UAE, which can be useful for prevention. We aimed to prospectively study pediatric and youth road traffic collision (RTC) related injuries, mechanism of collision, use of safety devices and clinical outcome in Al Ain City in order to give recommendations regarding their prevention.

## Methods

All injured children and youth 0–19-year-olds who were admitted to Al Ain City’s two major trauma centers or who died after arrival to these hospitals after being involved in a road traffic collision (RTC) from April 2006 to October 2007 were prospectively studied. A Research Fellow (HOE), one of the authors, interviewed the patients or caregivers on a daily basis and prospectively collected data. Variables studied included age, gender, nationality, crash mechanism, place of injury, road user type, position in the vehicle, use of safety equipment, location, time and season of the crash, anatomical body part(s) injured, severity, Revised Trauma Score (RTS), Glasgow coma scale (GCS), intensive care unit (ICU) admission, length of hospital stay, and outcome (survival or death).

Severity of injury of the affected body region was assessed by the Abbreviated Injury Scale (AIS), which divides the body into six anatomical regions and assigns each a severity including minor = 1, moderate = 2, serious = 3, severe = 4, critical = 5, unsurvivable = 6, and by the Injury Severity Score (ISS) [13], [14]. The ISS was calculated manually for each patient, using the Abbreviated Injury Scale Handbook, as the sum of squares of the three highest AIS scores from different body regions [15]. The revised trauma score (RTS) was derived from the GCS, pulse rate, systolic blood pressure, and respiratory rate obtained on arrival to the Emergency Department [14].

Using Statistical Package for the Social Sciences (IBM-SPSS version 19.0, Chicago, Il, USA), data were analyzed by regrouping, frequencies, and cross-tabulations. The Mann-Whitney U-test, Chi square test, or Fisher’s exact test were used as appropriate to compare continuous or categorical data of two independent groups. The Kruskal-Wallis non parametric test was used to compare severity of the main external causes of injury for more than two groups. A p value <0.05 was considered significant.

Incidence rates were estimated using 2005 census data, assuming the Al Ain population age structure to be similar to that for the entire Abu Dhabi Emirate [1]. Child and youth populations in Al Ain were estimated from national data, which found 11% of children and youth in the country to be in Al Ain [1]. Nationality was categorized into two groups – UAE nationals and non-UAE nationals, because studies have shown that traffic risks for UAE nationals differ from other nationalities, like driving sport utility vehicles, lower usage of restraints, speeding, and driving without a license [6], [7], [16].

Offending vehicles were classified as sedan (small vehicle), sport utility vehicle (SUV), or other vehicles (bus, light trucks, heavy trucks, and unknown). Some of the vehicle groups were very small to permit a reasonable analysis and we had to group them together.

The Local Ethics Committee of Al-Ain Health District Area has approved data collection for all road traffic collision trauma patients who were admitted to Al-Ain and Tawam Hospitals or who have died in the Emergency Department (UAE RECA/02/44). Data were collected prospectively on a specially designed hard copy form. Patients or children caregivers signed a written consent allowing using their anonymous data for research purposes.

## Results

### Personal Risk Factors: Gender, Age and Nationality

**Figure 1:**
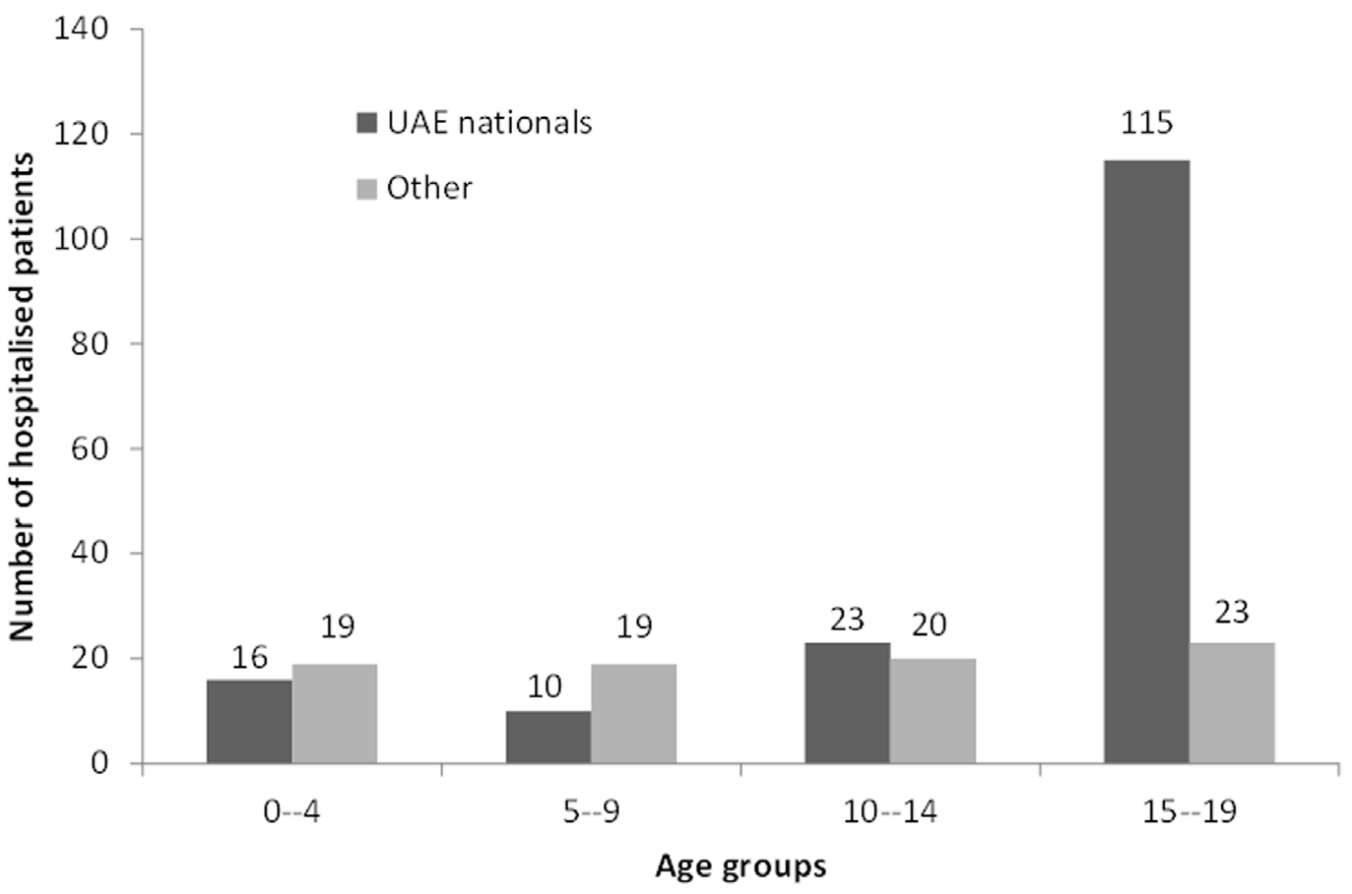
Traffic-related injury hospitalisations by nationality and age groups, Al Ain, 2006–2007 (n = 245).

There were 245 patients, 193 males (79%) and 52 females (21%), 0–19 years (mean age (SD) was 13.2 (5.8) years). All child and youth RTC trauma patients who were hospitalized during the study period were included in the study. Majority were UAE nationals (67%) and 15–19 years old (57%). (Table 1). With an estimated 108 967 children 0–19 years in Al Ain, the annual incidence of RTC hospitalizations was estimated to be 142 per 100 000 person-years. Higher incidence was among UAE-nationals and males, especialy in 15–19 years old (Table 1, Fig. 1). Although population ratio of UAE nationals to non-nationals was 0.7∶1, the traffic-related injury ratio was 2∶1, even higher difference was in 15–19 years olds UAE nationals comparing with non-nationals (5∶1). Males to females population ratio was 1.1∶1 and traffic-related injury ratio in this study was 3.7∶1.

**Table 1:** Traffic-related injury hospitalisations by road user type, nationality, gender and age group, Al Ain, 2006–2007 (n = 245).

Road user	Total		Nationality			Gender			Age group			
	n	%	UAE	Other	Ratio UAE/Other	Male	Female	RatioMale/Female	0–4	5–9	10–14	15–19
**Vehicle Occupant**												
Driver	48	20.0	42	6	7∶1	48	0	48∶0	0	0	1	47
Front seat	58	24.2	46	12	3.8∶1	51	7	7.3∶1	2	6	9	41
Rear seat	64	26.7	36	28	1.3∶1	35	29	1.2∶1	17	5	12	30
**Vulnerable road user**												
Pedestrian	36	15.0	10	26	0.4∶1	23	13	1.8∶1	11	16	7	2
Motorcyclist	23	9.6	21	2	10.5∶1	22	1	22∶1	1	0	5	17
Cyclist	11	4.6	6	5	1.2∶1	10	1	10∶1	2	1	7	1
**TOTAL**	240*	100**	161	79	2∶1	189	51	3.7∶1	33	28	41	138
**%**	100		67	33		79	21		14	12	17	57
**IR**	142		223	82		214	63		75	64	104	350

IR-incidence rate per 100 000 person years;

* 4 patients were other and 1 unknown; from them 3 were UAE nationals and 2 other nationality. There was missing information on road user type among 4 males and 1 female.

** Percents may not add to 100 due to rounding.

### Injuries by Type of Road User and Vehicle Type

Rear seat position was the most common (27%) position for vehicle occupants, followed by front seat (24%). More UAE nationals and males were injured in front seats as drivers or passengers (Table 1). Eight children under 10 years were injured in the front seat. Thirteen drivers (27%) were under 18 years old, the licensing age in the UAE. All pedestrians were injured by collisions with a car with majority children of other nationality (72%) and under 10 years of age (75%) (Table 1). More UAE nationals (91%) were injured as motorcyclist than non-nationals. Majority of motorcyclist were males (96%) and teenagers in 15–19 years of age (74%) (Table 1). Fifty one percent (90/175) occupants were injured in sedan cars, 44% (77/175) in SUVs and 5% (8/175) in other vehicles.

By comparisons of injuries among vehicle occupant vs vulnerable road users, vehicle occupants were significantly older and more UAE nationals (Table 2). Mortality among vehicle occupants was 4.1%. Percentage of ICU admission was higher among vulnerable road users and length of hospital stay was longer, although these differences were not statistically significant. There was no difference in Glasgow Trauma Score (GCS), Revised Trauma Score (TRS) and Injury Severity Score (ISS) between vehicle occupants and road traffic users (Table 2).

**Table 2:** Demographic and severity variables by road user type, Al Ain, 2006–2007 (n = 245).

	Vehicle occupant n = 170	Vulnerable road user(n = 70)	p-value
**Age (years)**	17 (2–19)	10 (2–19)	<0.001
**Gender (male)**	78.8%	78.6%	0.999
**Nationality (UAE national)**	72.9%	52.9%	0.004
**ICU admission**	19.4%	21.4%	0.1
**Length of hospital stay (days)**	3 (1–70)	4 (1–127)	0.38
**Mortality**	4.1%	0	0.11
**GCS**	15 (3–15)	15 (3–15)	0.95
**TRS**	12 (8–12)	12 (7–12)	0.64
**ISS**	5 (1–41)	5 (1–38)	0.64

p = Fisher’s Exact test or Mann Whitney test as appropriate; ICU = Intensive Care Unit, GCS = Glasgow Coma Scale, TRS = Revised Trauma Scale, ISS = Injury Severity Score.

Data are presented as number (%) or median (range) as appropriate.

### Crash Mechanism

The most common mechanism of injury was rollover of the vehicle (37%), followed by front impact collision (32%) (Table 3). There was a statistical significance in age (p<0.009) and GCS (p<0.024) between the different mechanisms of injury (Table 3). Front impact and side impact had lower GCS compared with rollover (mean (SD) of 13.31 (3.39) and 13.3 (3.65) compared with 14.27(2.42).Thirty two vehicle occupants were ejected during the crash, 9 were drivers, 9 front-seat and 14 back seat passengers. Sixty three percent of ejected persons (20/32) sustained a head injury with a mean AIS of 2.26.

**Table 3:** Demographic and severity variables by car crash mechanism, Al Ain, 2006–2007 (n = 245).

	Car crash mechanism				
	Front	Side angle	Rollover	Other	p-value
	n = 54	n = 32	n = 64	n = 21	
**Age (years)**	18 (2–19)	16 (1–19)	17 (2–19)	14 (2–19)	0.009
**Gender (male)**	83.3%	62.5%	79.7%	90.5%	0.074
**Nationality (UAE national)**	77.8%	65.6%	73.4%	71.4%	0.671
**ICU admission**	22.2%	21.9%	18.8%	19.0%	0.963
**Length of hospital stay (days)**	3 (1–42)	3 (1–73)	3 (1–70)	2 (1–36)	0.671
**Mortality**	5.6%	9.4%	1.6%	4.8%	0.279
**GCS**	15 (3–15)	15 (3–15)	15 (3–15)	15 (3–15)	0.024
**TRS**	12 (9–12)	12 (6–12)	12 (8–12)	12 (10–12)	0.885
**ISS**	5 (1–41)	5 (1–41)	5 (1–36)	5 (1–26)	0.535

p = Fisher’s Exact test or Mann Whitney test as appropriate; ICU = Intensive Care Unit, GCS = Glasgow Coma Scale, TRS = Revised Trauma Scale, ISS = Injury Severity Score.

Data are presented as number (%) or median (range) as appropriate; Mean (SD) of GCS was 13.31(3.39); 13.1(3.65); 14.27(2.42); 13.81(3.25) in front impact; side angle impact; rollover and other mechanisms respectively. Data are shown as median (range) in the table. Mann-Whitney test uses the ranks for analysis and that is why there is statistical significance between groups despite all groups have the same median range.

### Severity and Anatomical Location of Injuries

The most frequent anatomical location of injury was the head (42%) followed by extremities. Most severe injuries by Maximum Abbreviated Injury Scale (AIS) were to chest (mean AIS 2.68) and to spine (mean AIS 2.1) (Table 4). Fifty five percent (93/170) of vehicle occupants sustained head injury, with the highest percentage among rear seat passengers (61%; 39/64), followed by 56% of drivers (27/48 ) and 47% of front-seat passengers (27/58). From vulnerable road-users, 70% of motorcyclists sustained head injuries (16/23), 53% of pedestrians (19/36) and 36% of bicyclists (4/11). The frequency of head injuries was high in ejected patients (63%) and during roll-over crash (55%) (Table 5).

**Table 4:** Traffic-related injury hospitalisations by anatomical region and AIS severity, Al Ain, 2006–2007 (n = 558 regions).

Anatomical Region	Number & proportion of all injuries		Injury severity by Maximum AIS*			
	N	%	Mean	Median	Min	Max
**Head**	235	42	1.76	1	1	5
**Chest**	69	12	2.68	3	1	4
**Abdomen**	34	6	1.94	2	1	4
**Spine**	20	4	2.10	2	2	3
**Extremities**	175	31	1.80	2	1	3
**Superficial**	25	5	1.00	1	1	1
**Total**	558	100	1.87	2	1	5

* Maximum Abbreviated Injury Scale – only the most severe injury per body region was counted for each patient; Some patients have injury in more than one region.

**Table 5:** Number, proportion, and severity of traffic-related head injuries among vehicle occupants 0–19-year-old by car crash mechanism, Al Ain, United Arab Emirates, 2006–2007 (n = 171).

	Number			Head injury severity by AIS			
Car crashmechanism	All	Headinjury	%	median	mean	min	max
Front impact	54	26	48	2	2.38	1	5
Side impact	32	17	53	2	2.24	1	4
Rollover	64	35	55	2	2.17	1	5
Other	21	11	52	1	1.55	1	2
Total	171	89	52	2	2.17	1	5

AIS =  abbreviated injury scale.

### Place of Injury

Majority of traffic-related injuries, 67% (165/245), occurred on streets or roads, 22% (53/245) around homes in residential areas, 6% (14/245) off road, and 5% (13/245) in other locations. Seventy five percent (27/36) of pedestrians, 64% (7/11) of cyclists, and 26% (6/23) of motorcyclists were injured in housing areas. Forty eight percent of motorcyclists (11/23) were injured off road.

### Season, Day of the Week and Time of the Day

**Figure 2:**
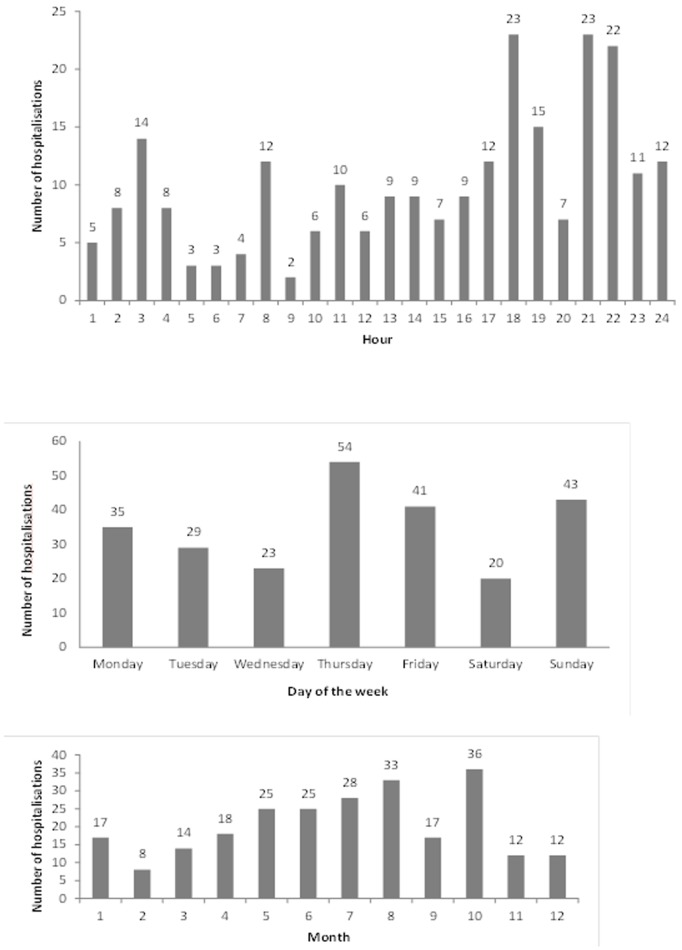
Traffic-related injury hospitalisations by time of collision (month, day and hour), Al Ain, 2006–2007 (n = 245).

Most of injuries occured during the summer (June–August); in the evening between 18∶00–24∶00, and on Thursdays which in the UAE is a last working day of the week similar to Friday in western countries (Fig. 2). Fifty eight percent of roll-over collisions (37/64) and 59% of front-collisions (32/54) happened at night 18∶00–6∶00.

### Use of Safety Equipment

Use of safety devices was extremly low. Only 3 (2%) vehicle passengers were using seatbelts. Small numbers did not allow us to make any statistical comparisons for seatbelt usage. There was no single child restraint in child car seat. Three motorcyclists used a helmet (13%) and 2 wore protective clothing (9%). No byclists used a helmet.

## Discussion

Traffic-related injuries are the leading cause of morbidity, disability and mortality worldwide [5], [17]. The fast economic growth of the country, dependent mainly on oil exports, have increased the construction of wide, fast-paced roads and number of cars used. For targeted injury prevention it is necessary to find the risk groups and other determinants. The estimated annual incidence of RTC hospitalizations among children and youth of less than 20 years was 142 per 100 000 person-years, even higher for UAE nationals (223 per 100 000), who were over-represented among our injured patients. More male UAE nationals were injured in the front of vehicle and as motorcyclists. Rollover was the most often crash mechanism, highlighting the need for use of restraints, which was minimal in our study.

The male preponderance in our study was similar to other studies [8], [17], [18]. Young male drivers have a higher average collision rate than women [19] and their death rate is double compared to females [20]. Risk-taking behavior, low use of safety belts and use of alcohol are some contributing factors for this outcome [21]. A high proportion of drivers (27%) in our study, all males and majority UAE nationals, was under 18 years of age. Eighteen years is the legal age for achieving a driving license in the UAE. These young drivers can be used for family affairs without considering the related risks. The proportion of young UAE-national women drivers in the UAE is low mainly because of cultural reasons.

We included all children and youth 0–19 using the WHO classification of 5 years age categories, although UN Convention on the rights of the child is using age <18 [5]. Using same categories as WHO is important for international comparisons of mortality and morbidity. Similarly UNICEF recent report “The state of the World’s children 2011” used the age including 19 years dividing into early (10–14) and late adolescent age (15–19) [22].

Most of our patients were injured in the evening time, similar to other studies [18]. Injuries decreased from Sunday (first working day) during the week with sharp increase on Thursday (last working day before the weekend) and during summer months, when children have holidays (July and August). Another month with frequent injuries was October, during data collection period it was a month of Ramadan, when the risk of RTCs is higher [23].

Although the proportion of head injury among child and youth vehicle occupants (55%) was similar to other studies from the region [18], [24], this proportion was almost double when compared with countries having a higher frequency of use of restraints (such as Australia) [25]. Not suprisingly the highest proportion of head injury was among rollover crashes (55%). Persons ejected from cars during front or side impact, or rollover sustained even higher proportion of head injury (63%). The severity of injury will increase when vehicle occupants are ejected from the vehicle [26]. Rollover of the vehicle was a common car crash mechanism in our study. There are different possible explanations for this: a) many of cars, driven by UAE nationals are SUVs, having a higher center of gravity. This leads to imbalance on curved roads when driving with high speed. b) There are many 3 lanes roundabouts in Al Ain, where fast driving cars tend to hit the side of the road. c) A high popularity for desert and other off-road driving. This even increases the importance of using safety restraints in our community.

Previous studies in the UAE has shown a very low frequency of use of safety restraints [7], especially among UAE nationals [6]. Only three passengers (2%) in our study used seatbelts. There is evidence that use of seat belts in adults can reduce fatalities, injury severity, and healthcare costs [27], [28], [29]. The use of normal seat belts is recommended by WHO for children over the age of 10 years or above 150 cm in height [5]. Other child restraint systems, as rear- and forward facing seats for infants and toddlers or booster seats for older children, if appropriately used, are very effective at preventing fatalities [5], [30]. Persisting misconception, that unbelted vehicle occupant can escape safely from the car is common among young UAE nationals [6]. Unbelted occupants are more likely to be ejected from vehicles during collision [6], with a 70% increase in mortality compared with belted [31].

Mandatory seat-belt legislation, if properly enforced, can save many lives. For example, when Victoria (Australia) implemented the mandatory seat belt legislation in 1971, the annual number of car occupant deaths had fallen by 18% in the end of that year, and by 26% in 1975 [32]. In the United Kingdom in 1979, front seat belt use increased from 37% before the law to 95% after the law. This was associated with 35% reduction in traffic-related hospital admissions [33], [34].

Despite the awareness of the value of safety restraints is growing in the Gulf Cooperation Council (GCC) countries, compliance still varies between these countries. We have compared the GCC countries with another 37 high-income countries regarding seatbelt compliance and road traffic death rates [35]. The median seat belt compliance for the GCC countries was 48% compared with 85.5% for the other high income countries. The estimated road traffic death rate in the GCC countries was more than double that of the other high income countries [36]. Using data from 46 high income countries, Abbas et al. [36] have demostrated a very highly significant negative correlation between seatbelt compliance and road traffic fatalities.

A proper use of child restraint reduces risk of fatality among infants by 71% and among toddlers by 54% [37]. Elliot et al. [38] showed that use of appropriate child restraints among children aged 2 to 6 years, reduced the risk of mortality by 28%. Although there was a recent attempt of introducing the child car seat legislation in the UAE, it is still not properly enforced [2].

Head injury is a major contributor for death of RTC. We have previously shown that low Glasgow Coma Scale was the most imporant predictor of mortality [39]. Head injury will increase in unrestrained vehicle occupants [26]. Head injury among motorcyclists was very common. Despite legislation, the usage of motorcycle helmet was extremly low (13%). Helmets reduce the severity of head injury, morbidity and mortality [17], [40], [41], [42]. Helmet use varies from almost non-existent in some low-income countries to almost 100% in states with proper legislation enforcement [17]. Nevertheless, they do not provide sufficient protection if un-certified models were used [43]. The licensing age for motorcyclists in the UAE is 17 years [16]. Despite that 14 motorcyclists were younger than 17 years in our study, majority were UAE nationals. This may be explained by the fact that many recreational motorcyclists are young UAE nationals, while most riders who use motorcycles for work or transport are older non-nationals [16]. There is evidence in the literature supporting increasing the age of licensing to protect against motorcycle injury [44].

The most frequently injured vulnerable road users in our study were pedestrians (15%). Nevertheless, this was less compared with other countries [5], [45]. The patient’s age, vehicle design and impact speed are risk factors of pedestrian injury severity and mortality [46]. Surprisingly, non-nationals had higher risk of pedestrian injury in our study, suggesting that unsupervised children played in the housing areas in proximity of driveways or around parked vehicles. Higher percentage of vulnerable road users was admitted to the ICU and they stayed longer in the hospital compared with vehicle occupants.

### Limitations of the Study

Our study did not include the more severely injured patients who died on the scene before arriving to the hospital and those with mild injuries who were treated at primary health care facilities or in the Emergency Department. Furthermore, our data were from a limited time research project (2006–2007). This may not reflect exactly what happens at present as many changes occured during the last six years. There are no recent data available for comparisons. Collected information about collision, like place of injury, type of road user, use of restraint etc. was self-reported, having its own limitations.

### Conclusions

Male drivers and UAE nationals were at high risk of RTC as drivers and as motorcyclists. Ejection rate was high because safety restraint use was extremely low in our community. More education and law enforcement focusing especially on car/booster seat use is needed.
